# Family Caregiver Perspectives on Digital Methods to Measure Stress: Qualitative Descriptive Study

**DOI:** 10.2196/66034

**Published:** 2025-04-24

**Authors:** Louise Rose, Sian Saha, Emily Flowers, Chee Siang Ang, Alexander J Casson, Joan Condell, Faith Matcham, Tony Robinson, John Rooksby

**Affiliations:** 1 Faculty of Nursing Midwifery & Palliative Care King's College London London United Kingdom; 2 School of Computing Kent and Medway Medical School University of Kent Kent United Kingdom; 3 Department of Electrical and Electronic Engineering University of Manchester Manchester United Kingdom; 4 Faculty Of Computing, Engineering & Built Environments University of Ulster Derry United Kingdom; 5 School of Psychology Sussex University Sussex United Kingdom; 6 Department of Computer and Information Sciences Northumbria University Newcastle United Kingdom

**Keywords:** psychological stress, qualitative research, family caregivers, digital stress monitoring, caregiver burden, biosensing technologies, descriptive study, qualitative study, semistructured interview, ecological momentary assessment, remote monitoring, smartwatches, fluid biosensors, framework approach, wearables, digital technologies, digital health

## Abstract

**Background:**

Family caregivers provide essential care in the home to millions of individuals around the globe annually. However, family caregiving results in considerable burden, financial hardship, stress, and psychological morbidity. Identifying and managing stress in caregivers is important as they have a dual role in managing their own health as well as that of the person they care for. If stress becomes overwhelming, a caregiver may no longer be able to perform this essential role. Digital methods of stress monitoring may be 1 strategy for identifying effective interventions to relieve caregiver burden and stress.

**Objective:**

This study aims to explore the perceived acceptability, challenges, and opportunities of using digital and biosensing technologies to measure caregiver stress.

**Methods:**

We conducted a descriptive qualitative study using semistructured interviews with an interview guide structured to obtain qualitative data addressing our study aims. We used reflexive thematic analysis methods. We recruited adult family caregivers (aged 18 years and older) currently or previously caring for an adult in the home with significant health issues. Interview questions focused on stress monitoring more generally and on ecological momentary assessment, remote monitoring technologies such as smartwatches, and fluid biosensors.

**Results:**

We recruited 27 family caregivers of whom 19 (70%) were currently in a caregiving role, and the remainder were previously in a caregiving role. We identified 3 themes with 10 subthemes addressing elements of acceptability, challenges, and opportunities of using digital and biosensing technologies to measure caregiver stress The themes comprised “providing meaningful data” with subthemes of “monitoring without action is pointless,” “monitoring that enables self-management,” and “seeing the bigger picture”; “low-burden monitoring” with subthemes of “low effort,” “practical alongside daily routines,” and “retaining control over monitoring”; and “inadvertent harms of stress monitoring” with subthemes of “stigma of stress,” “need for discretion,” “contributing to stress,” and “trust.”

**Conclusions:**

In this descriptive qualitative study examining the perspectives of a diverse sample of family caregivers on methods of stress monitoring, we identified 3 themes addressing elements of acceptability, challenges, and opportunities. These provide useful considerations for the use of stress monitoring and implementation of interventions to ameliorate family caregivers’ stress of relevance to social care and community teams, researchers, and policy makers. These include providing meaningful situationally specific data resulting in action, that does not contribute to caregiver burden, or inadvertent harm to either the caregiver or the care recipient.

## Introduction

Family caregivers provide essential care in the home to millions of individuals around the globe annually resulting in substantial cost savings to public and private health care systems [1]. Family caregivers provide unpaid care, generally without formal training, to a relative with a chronic illness or disability, enabling them to remain at home [2]. This promotes quality of life for the care recipient and maintains family bonds and relationships which also benefits the caregiver. However, family caregivers, who frequently have other responsibilities such as work and parenting, may experience considerable chronic stress, psychological morbidity, higher cortisol levels, lower immune responses, physical illness, and financial hardship due to the physically and psychologically demanding nature of caregiving responsibilities [3,4]. These negative consequences of caregiving are frequently hidden and underrecognized resulting in a lack of dedicated support [5].

Caregiver stress is defined as a state of mental or emotional strain or tension resulting from demanding circumstances associated with caregiving [6-8]. Numerous interventions designed to reduce caregiver stress have been developed and evaluated. Examples include psychosocial interventions delivered by health care professionals [9], in-person peer-support groups [10], and printed informational materials [11]. More recently, interventions have incorporated digital elements, giving in-person access to support is frequently challenging considering the high burden of caregiving responsibilities [12]. Examples include mindfulness apps [13], online support groups [14], and web-based information sources. This was expedited by the COVID-19 pandemic during which the time to adopt digital technologies went from 8 years to approximately 2 weeks [15]. However, few in-person or digital interventions have demonstrated consistent success in reducing caregiving-associated stress, psychological morbidity, and caregiver burden [16,17]. Furthermore, the identification of stress that is impacting caregiver well-being and ability to provide care poses challenges. This is due to its subjective and dynamic nature influenced by numerous mediators as well as the stigma attached to admitting vulnerability and the need for support [17].

One reason for the variable effects of interventions to support family caregiver well-being could be as a result of the outcome measures used in randomized controlled trials [18]. Psychological outcomes such as anxiety and depression (eg, Hospital Anxiety and Depression Scale [19]), health-related quality of life (eg, EQ-5D-5L [20]), and caregiver burden (eg, Zarit Burden Interview [21]) are most commonly collected using self-report measures measured at baseline and then discrete timepoints during or on completion of the intervention. The use of these measures at fixed time points is subject to recall and social desirability bias [22]. They also may not sufficiently capture the physiological and psychological responses to caregiving stressors in real-time, which may be more responsive to interventions to reduce caregiver burden.

There are several digitally based alternatives and complements to traditional self-report questionnaires. Ecological momentary assessment (EMA) is a measurement method that can capture digital questionnaire response data in real-time, in real environments, and over time, thus avoiding recall bias [23]. EMA can help to identify subtle nuances in changes in mood or the presence of confounding or mediating stressors. A recent systematic review of EMA in family caregivers [24] identified 12 studies recruiting only 461 participants of predominantly care recipients with Alzheimer or dementia indicating a limited evidence base and a gap in research in other caregiver populations. Remote monitoring technologies (RMT), including data passively collected from smartphones and wearable devices can enable real-time, longitudinal tracking of well-being by active collection of data from questionnaires and/or by collecting physiological and behavioral data [25]. Passive RMT can collect digital biomarkers of caregiver stress, for example, heart rate from photoplethysmography sensors and activity from accelerometry sensors indicative of sleep and physical activity patterns [26]. It can also collect data on digital behaviors such as phone calls and messaging behaviors, use of apps, and response to notifications that might inform understanding of caregiver stress management methods [27]. However, studies of RMT generally focus on monitoring the care recipient and how this can support family caregivers, not monitoring caregiver stress [28]. Wearable electrochemical biosensors for in situ analysis of body fluid such as sweat may also offer the opportunity to provide continuous real-time physiological information [29,30].

Given the challenges of identifying effective interventions to reduce stress and psychological morbidity of family caregivers, the potential of newer methods of monitoring, and the paucity of evidence using these techniques in family caregivers, we sought to explore perceived acceptability and challenges of using digital (EMA and RMT) and fluid biosensing technologies to measure caregiver stress. We intend these data to inform the development of novel digital strategies to effectively identify and manage stress suitable for a heterogeneous family caregiver population.

## Methods

### Study Design

We conducted a descriptive qualitative study using semistructured interviews. We adopted a constructivist and relativist stance to explore the influence of different experiences of participants on their interpretations of using technology to measure caregiver stress. We adhered to the Consolidated Criteria for Reporting Qualitative Research (COREQ) guidelines during manuscript preparation (Multimedia Appendix 1) [31]. The study team was comprised of senior and early career researchers (both male and female) with health care, computer science, and engineering experience, none of whom have personal family caregiver experience, but some (LR, SS, and EF) with previous and ongoing experience working with family caregivers of individuals with motor neurone disease and survivors of critical illness.

### Participants

We included adult primary family caregivers (aged 18 years and older) currently or previously caring for an adult in the home with health issues. We did not apply a time restriction in terms of how long-ago caregiving occurred. We excluded those with a caregiving role for a healthy child (ie, parent, teacher, or caretaker role) and those with a professional (paid) caregiving role.

### Recruitment

We used a multimodal recruitment strategy. This included contacting previous study participants participating in a digital peer support program for caregivers of people with motor neurone disease [32], snowball sampling, social media (X, formerly known as Twitter), patient charities (ICUSteps and Motor Neurone Disease Association), the King’s College London weekly research volunteer e-circular, and through E-Carewell (research program of digital support tools for carers run by Ulster University). Although we used convenience sampling in that we interviewed all caregivers meeting our inclusion criteria that responded to our recruitment methods, we did seek diversity in terms of age, sex, ethnicity, and relationship to the person being cared for through our multimodal recruitment methods.

### Interview Guide and Data Collection

We iteratively developed our interview guide with pilot testing during the first interview (Multimedia Appendix 2). Questions explored digital methods to measure caregiver stress generally and more specifically the use of EMA, RMT, and biosensors to measure stress.

We conducted one-on-one interviews (from February 2023 to August 2023) over video using Zoom (Zoom Video Communications), or telephone call, depending on participant preference. Interviews were conducted by a nurse researcher (SS) with interview training and experience. SS had no prior relationship with participants, although some participants were previously interviewed following their participation in our digital peer support trial. Interviews were digitally recorded, professionally transcribed, and anonymized. Interview reflexive notes of the interviewer’s general impressions and thoughts were made during interviews. Transcripts were not returned to participants for data verification due to concerns about participant burden in addition to existing caregiver burden. Themes and subthemes were discussed with former caregivers participating as peer supporters in another ongoing study. No participants withdrew their data. We continued interview recruitment until the study team considered that no new data was being identified, and we had reached sufficient information power [33] for our relatively narrowly focused aim, participant specificity, and quality of dialogue while also not specifically conducting deductive analysis using theory and using a cross-case analysis strategy (Multimedia Appendix 3).

### Data Analysis

We conducted the 6 steps of Brain and Clark reflexive thematic analysis [34]. We followed the following procedures: (1) familiarization with the interview data, (2) generating initial codes, (3) generating initial themes, (4) reviewing themes, (5) defining and naming themes, and (6) writing up.

To enhance trustworthiness, transcripts were read and reread from March 2023 following the first interview to March 2024 with an initial code developed independently by 2 research team members (SS and EF) using the comment and highlight functions of Microsoft Word and then charted using Microsoft Excel. Two other team members, including the principal investigator (LR) and co-investigator (JR), reviewed and performed initial coding of 40% of the transcripts to gain familiarity with the data and to guide further coding discussions. Coding and deductive theme and subtheme development were then discussed iteratively over an extended period by the core team (SS, EF, LR, and JR) at monthly meetings. This process of iterative discussion, sense-checking, and rereading of transcripts meant agreement was reached that the themes and subthemes fit with the data without specific conflicts of opinion. Themes were reviewed, discussed, and approved by the wider study team (JC, FM, JA, AJC, and TR).

### Ethical Considerations

We obtained research ethical approval from King’s College London Minimal Risk Committee (reference: MRA-22/23-34473). All participants were informed of the study’s objectives, their rights as participants, and the confidentiality of their responses. Written or verbal informed consent was obtained before the interview. We anonymized all data following transcription and the audio files were deleted. Participants were given a £15 (US $19.45) gift voucher in lieu of their time.

## Results

### Study Participants

We recruited 27 caregivers to participate, with interviews lasting between 15 and 49 minutes. Most 19 (70%) were currently, as opposed to formerly (8, 30%) in a caregiving role, with 15 (56%) of the 27 caregivers indicating that they delivered between 10 and 24 hours of care per day. Recipients of care provided had a range of conditions with the most common being motor neurone disease, recovering from admission to intensive care, or dementia (Table 1).

**Table 1:** Demographics of interview participants (N=27).

Characteristics		Participants, n (%)
**Age (years)**		
	18-40	2 (7)
	41-65	17 (63)
	Older than 65	8 (30)
**Sex**		
	Male	8 (30)
	Female	19 (70)
**Ethnicity**		
	Asian or Asian British	2 (7)
	Black, African, Caribbean, or Black British	3 (11)
	White British	19 (70)
	Mixed or multiple ethnic groups	2 (7)
	Other ethnic groups	1 (4)
**Education level**		
	Completed secondary school education	5 (19)
	Some tertiary (college or university) courses	2 (7)
	Completed undergraduate level qualification	13 (48)
	Completed postgraduate degree (master’s and higher)	7 (26)
**Employment**		
	Retired	10 (37)
	Full-time work	10 (37)
	Self-employed	6 (22)
	Part-time or casual work	1 (4)
**Care recipient**		
	Spouse	11 (41)
	Sibling	3 (11)
	Parent	11 (41)
	Grandparent	1 (4)
	Grandchild	1 (4)
**Primary patient health needs**		
	Motor neurone disease	9 (33)
	Intensive care survivor	6 (22)
	Dementia	5 (19)
	Learning difficulties	3 (11)
	Others^a^	4 (15)
**Still in a caring role**		
	Yes	19 (70)
	No, previous caregiving role	8 (30)
**Number of caregiving hours**		
	10-24 hours/day	15 (56)
	5-30 hours/week	4 (15)
	1 hour/week	1 (4)
	Variable depending on need	6 (22)
	No data	1 (4)
**How long caring (years)**		
	Less than 1	4 (15)
	1-5	14 (52)
	5-10	5 (19)
	More than 10	4 (15)

^a^Others comprise frailty, Parkinson disease, and stroke.

We identified 3 themes with 10 subthemes (Table 2). The 3 themes were “providing meaningful data,” “low-burden monitoring,” and “inadvertent harms of stress monitoring.” in the later sections, we discuss these themes and their subthemes as they applied to the more general concept of using technology to monitor stress and more specifically to EMA, RMT, and fluid biosensors.

**Table 2:** Themes, description, and subthemes.

Theme	Description	Subthemes
Providing meaningful data	Monitoring should provide situationally specific data resulting in action to ameliorate stress, whether it be from an external source or by themselves.	Monitoring without action is pointless Monitoring that enables self-management Seeing the bigger picture
Low-burden monitoring	Does not add to the existing high levels of caregiver burden, that is, low effort, flexible, and practical in the real world of caregiving.	Low effort Practical alongside daily routines Retaining control over monitoring
Inadvertent harms of stress monitoring	The harm that might arise given the stigma associated with stress, the impact on the care recipient in terms of self-perceived burden, the detrimental effect of being aware of high-stress levels without the ability to access support or enact self-care due to caregiving responsibilities, and having trust in data safety.	Stigma of stress Need for discretion Contributing to stress Trust

### Theme 1: Providing Meaningful Data

The theme of “providing meaningful data” was consistent throughout the dataset and directly addresses our research aim of understanding the acceptability of novel methods to measure caregiver stress. Participants emphasized monitoring would not be meaningful and thus considered unacceptable without an accompanying action from an external source such as a health or social care team or enabling self-management strategies. Monitoring data had to be readily interpretable and meaningful to them to be able to enact self-management. Participants also emphasized that meaningful, and thus acceptable, stress monitoring required an understanding of the “bigger picture” that would include fluctuations in stress over time in response to stress mediators as opposed to a snapshot view.

#### Subtheme 1: Monitoring Without Action Is Pointless

Most, but not all, participants considered the use of stress monitoring devices when data were measured but not shared as unacceptable in research or daily real-life contexts.

I don't think I'd be very happy in wearing it and then the results just went off somewhere and I didn't know what it said. I'd like to know.

ID10

Participants indicated the need for a response from an external health or social care provider when experiencing high-stress levels as detected by monitoring as they may not be able to self-manage given their level of caregiver activities and corresponding burden. This would provide a psychological safety net with reassurance that someone was keeping an eye on them, their well-being mattered, and that support would be in place when caregiving stress became overwhelming. Monitoring without access to such a response was viewed as pointless.

I would actually be able to think,… there’s someone keeping an eye on this and if I’m moving out of the amber and I’m going into the red zone, someone’s going to notice… at that point someone might just call up and say how are you doing?

ID1

When asked about RMT, participants reiterated their concerns about measuring stress that was not paired with an intervention to reduce stress and therefore, could not help to reduce stress. Participants also recommended that stress data should be given to the caregiver being monitored alongside expert interpretation and advice to understand how to manage it appropriately.

Measuring is fine and finding out all of that, but what do you do about it? What do you put in place? That's what people want.

ID17

#### Subtheme 2: Monitoring That Enables Self-Management

Participants were keen to receive stress data with an objective measure that they could interpret such as a numeric score to then take actions to ameliorate their stress such as deep breathing.

You can see on a scale of one to ten and then,… if you're a six or an eight and then a reminder to abdominally breathe or whatever, or maybe some guidance on how to do it.

ID21

Participants also thought measuring stress would help them to identify triggers or particularly stressful situations that would help guide actions to relieve stress.

It might help then if you know when something's happened that has made you more stressed, then you might be able to cope with it a bit better if you're aware of that situation really.

ID10

Many participants expressed that without these objective measures, they would not otherwise know when they were stressed as they did not recognize other outward manifestations such as getting angry or frustrated as stress.

Sometimes you don't recognise your stress, because you're just that long... Because you're having stress all the time, you don't always recognise it.

ID26

When asked about EMA, participants appeared to either really like the concept or not like it at all, depending on how they coped with their own stress. EMA appealed to those participants who liked to reflect on their experiences and found value in documenting them or communicating them to others.

Yes, actually that would be really helpful, because I think sometimes it's not having a moment to just stop and actually work out, how am I feeling?

ID22

When asked about RMT, participants thought being aware of objective stress data would enable positive action to reduce stress levels and improve well-being.

So something like that would be good because if I could see something, say my heart rate going up, and I may be sitting on the sofa,… that may prompt me to do something more about it.

ID23

#### Subtheme 3: Seeing the Bigger Picture

Many participants liked the idea of frequent or continuous stress monitoring as they recognized that stress varies in response to triggers and mediators, which may be unpredictable due to the nature of their caregiving situation. Therefore, frequent or continuous monitoring would help to capture the fluctuating nature of their stress.

…like if it was yesterday then the answers would have been fundamentally different from the day before.

ID19

Some participants thought the “snapshot” of data enabled through stress monitoring, particularly EMA, might not be representative of overall well-being and therefore, not meaningful for capturing the impact of stress in the longer term.

### Theme 2: Low-Burden Monitoring

#### Overview

The theme of “low-burden monitoring” was consistent throughout the dataset and in all monitoring modalities. This theme addresses our research aim of understanding the potential challenges of stress monitoring and ways to overcome these challenges to make stress monitoring more acceptable. Participants spoke extensively about the importance of not adding to the existing high levels of caregiver burden through stress monitoring. They emphasized that any form of stress monitoring must be low effort, flexible, and practical in the real world of caregiving.

#### Subtheme 1: Low Effort

When asked about EMA delivered to their smartphone, watch, or tablet, participants liked that this form of questioning would be simple to understand and quick and easy to complete.

I think the key to that it’s always got to be short and sharp… I think simplicity is key.

ID16

Most participants liked the idea of scoring their stress but wanted this to be minimal effort. Numeric scores (eg, 1 to 10) or visual scales (eg, faces displaying different emotions or traffic light colors) were considered easy to understand and quick to complete. Participants recommended this could include a short text optional follow-up allowing caregivers to communicate the nuances of their answers.

I’ve got a diary, you get the face and you put whether you’re smiley face, sad face… so ticking one of those as to what kind of day you’ve had and just even a sentence or bullet points as like in a box to just write your own words, that would be definitely something I would have used and found useful.

ID9

Participants who were less favorable of EMA perceived they would feel burdened or irritated as these questions interrupted their day resulting in increased stress levels. However, participants identified that not responding to EMA questionnaires could recognize stress was present and therefore an equally important outcome.

I suppose if you're feeling stressed and it kept coming through, you'd probably go, “Leave me alone. I can't cope with this.” But that's probably a good way of finding out when people are stressed at different times of the day.

ID10

When asked about RMT, all participants had prior knowledge of RMT with an understanding of their ability to measure vital signs and activity metrics such as steps. Participants generally perceived this to be of low burden, offering an easy solution to stress monitoring.

It's just the idea of how like technology's helping us eternally without us actually physically doing things like going to a doctor to tell us how's that stress or talking to someone. It's something you are doing like in a very comfortable, easy manner.

ID2

Those participants who owned or had used RMT considered stress monitoring as an appealing addition to current device functionality.

Yeah, because I wear that all the time anyway,… great for measuring your heart rate and your steps and that too… I suppose if that was able to pick up stress in some way, that would be actually good as well too.

ID27

Some participants who did not own RMT also had positive perceptions but with the caveat that it would have to be a low burden to them.

If there was a specific reason for it, and I was helped very, very clearly to set the thing up in the first place, that might be a very useful thing.

ID17

Other participants with no experience of RMT were less positive, considering them to be unnecessary or complex to set up and keep charged.

I know my level of fitness, I know when I’m not performing at that level, I don’t need a Fitbit to tell me that I need to either work harder.

ID1

When asked about fluid biosensors, interestingly participants considered these more appealing than EMA or RMT given their simplicity.

When they got to the patch [stress sensor], I thought, I think this is the best one of all of them… I think because it was so maybe it sounded simpler.

ID27

#### Subtheme 2: Practical Alongside Daily Routines

Any form of stress monitoring was viewed as needing to be comfortable, enable sleep, and be low maintenance and practical in any situation so it could be worn and forgotten, thereby not contributing to caregiver burden given the physically and emotionally demanding nature of caregiving.

I guess the key is that it be low maintenance, shouldn’t be another thing to do so it doesn’t turn into something else you then have to do, add to your long list of things to do.

ID16

When asked about EMA, some participants doubted whether they could engage due to practical difficulties such as the ability to navigate the technology or pragmatics as to whether they had their smartphones with them throughout the day.

I don't know that I would actually be answering them in a timely way. I usually have the phone somewhere around me, but you know what it's like. We don't have the correct pockets in our clothes, for a phone falls out or something.

ID15

When asked about a fluid biosensor, other practicalities raised included problems with airport security scanners, being waterproof to allow washing, and challenges with adherence issues relating to body hair.

Only if you’re going through airport securities and things like that. It would have to be shower-proof or easily or on a chain that you can just take off or something, rather than actually stuck to you maybe. You’re not sticking anything like that on me, I’ve got too much hair.

ID12

#### Subtheme 3: Retaining Control Over Monitoring

Enabling control and flexibility in responding to and reviewing stress monitoring was considered important in reducing burden, particularly in association with EMA questionnaires. Enabling control included monitoring frequency, that is, when they would answer the questions and how often, adapting monitoring requirements to changing caregiving or other situations, and monitoring that represented minimal intrusion to their caregiving and other activities. Participants expressed that stress monitoring that addressed these aspects could lead to a more personalized monitoring approach that would make them more inclined to engage.

If it was built and designed in a way that wouldn’t make you feel like, oh my God this is another thing that I have to get done, if it was felt that it was optional and if you didn’t fill in for one day, then that’s not the end of the world kind of thing.

ID16

Participants also considered RMT offered flexibility over when someone in a caregiving situation could pay attention to their stress levels.

I think if it was recorded so I could look at it at a later time, because I think I would want to just put it on and not think about it. So then maybe look at the results of the day at the end of the day when you had that 10 minutes of quiet.

ID9

### Theme 3: Inadvertent Harms of Stress Monitoring

#### Overview

The theme of “inadvertent harms of stress monitoring” was distinct from that of the theme “low burden” but again was consistent throughout the dataset and to all monitoring modalities. This theme revealed further potential challenges that might be less immediately obvious to researchers and health care professionals than those identified in the “low-burden theme.” Participants were concerned about other potential inadvertent harms of monitoring themselves that might arise from the stigma of stress, the impact on the care recipient and the associated need for discretion, and how being aware of high-stress levels might further contribute to caregiver stress when unable to access support or enact self-care due to caregiving responsibilities.

#### Subtheme 1: Stigma of Stress

Participants were concerned about the potential harm of visible monitoring that would draw the attention of others and would provoke questions. Visible monitoring was considered likely to draw unwelcome attention that something was different about them, or imply they were not coping thus inducing negative reactions such as shame.

You get all sorts of stupid questions. Like, “Why are you wearing that for? Well, I'm being monitored in case I go crazy.

ID3

When asked about EMA, participants felt that the perceived stigma associated with being a caregiver and being stressed and therefore not coping might lead to providing answers to EMA questions reflective of social desirability bias rather than their true situation. As well as providing ratings to others that may not represent their situation, participants identified that they may not be able to admit to themselves their true feelings relating to caregiver stress and therefore would provide responses that were nonreflective.

I doubt my own self-assessment, that evaluation, I doubt I'm being totally honest and truthful with myself and then I just choose a number on that scale.

ID4

When asked about RMT and fluid biosensors, participants considered that this objective stress data from physiological measures as opposed to subjective EMA self-report was important given the tendency to put on a brave face.

It would eliminate where people are just being nice, because they don't want to say that they're not coping or they're not stressed over anything… I think that you'd get a lot of data from that.

ID24

#### Subtheme 2: Need for Discretion

Participants expressed a strong recommendation for stress monitoring to be discreet from the care recipient, other family members, friends, and members of the public. This arose from a desire to protect their family members from feeling guilty about their care needs and the associated caregiving burden being the cause of their family caregiver’s stress.

I don’t think something like that will contribute any benefit to the psychological wellbeing on the patient... They feel they’re a burden already, and now the burden is being measured externally, if it was invisible, if it was something strapped to the chest or whatever, yeah, but certainly not something that was visible.

ID8

Participants also thought that visible stress monitoring may upset others in the same way a scar or body disfigurement might.

Something that isn't going to look out of place or be conspicuous in any way…. I'm not fussed about showing it, but I don't want it to upset someone else.

ID7

Some participants thought visible stress monitoring could result in them thinking about their stress all day and so wanted any device to be discreet so that they could forget about it.

You'd almost want to be able to wear it to sort of try and forget about it as well rather than be too aware that it's there and thinking, ”Oh, there is this... What's this showing up?

ID10

When asked about EMA, participants were concerned that questions popped up on their devices or their answers could be seen by others, particularly the person being cared for.

If it came through the SMS text system for example, I think that could be quite dangerous because you know, people will share iPads, they share phones, they pass each other their phones to show photos to one and other and then suddenly if a pop up comes up…. that can be damaging to a relationship.

ID14

When asked about RMT, although there remained the need for discretion, RMT was thought to be easily disguised as a timepiece and therefore, raised less concern than other forms of stress monitoring.

A watch maybe, but Fitbits, they’re a type of, you can identify that kind of thing, it’s for purpose. A watch could be telling the time. So yeah, that kind of device I would go with.

ID8

When asked about fluid biosensors, because this sensor technology was considered novel, participants stressed even more the need for it to be discrete and not provoke questions from others. Participants would only be happy to wear the biosensor if hidden from view.

I think if it was something that was stuck to your skin so that it's out of sight, then I don't think I'd be too bothered as long as it wasn't obviously too big.

ID10

#### Subtheme 3: Contributing to Stress

When asked about EMA, participants were concerned about the effect of receiving questions about stress when in a highly stressed situation. They suggested that questions must be carefully worded to ensure the questions themselves did not provoke further stress.

It would be a bit dangerous to say anything about are you feeling stressed? Are you under pressure? Because that might make them feel they are or that you think they are and so on, so you would have to keep it bland.

ID12

When asked about RMT, there were opposing views about whether being aware of the data could be beneficial or harmful. Some participants thought that being aware of stress data would cause additional stress through people getting obsessed with it or misinterpreting it.

Forever watching the resting rate, how many steps a day, it drives me around nuts because I'm forever looking at it… I get overly stressed because I look at it too much.

ID6

#### Subtheme 4: Trust

When asked about general concerns, there was very little participant discussion around data privacy and security. Most, but not all, participants expressed trust in RMT and considered that the data collected were handled in a secure manner.

I guess I'd assume that the information's going into a secure place and will be handed off very sensitively, and so, yes, I have all assurances around that… that would be interesting, and it's evidence-based.

ID22

I am very, very, concerned about my privacy especially with technology. And the more I hear about, amazing developments like AI for instance, it quite terrifies me in some aspects. I know it can be very, very beneficial. But I would just need to know what it's doing.

1D17

When discussing fluid biosensors, participants expressed more concern about the collection of data without their knowledge or informed consent.

Maybe if I had a very detailed list of what information that would be collated through the sensor…. There's a perception that the Fitbit is gathering specific data, but the sensor might be able to gather all sorts of data, some which might be helpful in terms of measuring stress, but other data that isn't necessarily relevant, but it's been collected anyway.

ID22

## Discussion

### Principal Results

In this descriptive qualitative study, we sought to explore the perceived acceptability and challenges of using digital and biosensing technologies to measure caregiver stress. Our key findings were that family caregivers considered any form of monitoring should provide meaningful situationally specific data resulting in action to ameliorate stress, whether it be from an external source or by themselves. Stress monitoring should not add to existing high levels of caregiver burden by being low effort, practical alongside daily routines, and enabling caregivers to have some control over how they respond to monitoring. Monitoring also should not inadvertently result in harm or cause additional stress given the perceived stigma associated with stress that could indicate failing to cope or cause the recipient of care to feel guilty or burdensome as the cause of their caregiver’s stress.

The finding that stress monitoring, irrespective of digital or other formats, needs to be linked with actions to ameliorate stress is not unique [35]. However, this is particularly poignant for caregivers given they typically have very little time for themselves due to their caregiving responsibilities and frequently deprioritize their own needs [36]. This means stressful self-management actions may be deferred or not attempted at all, and external sources of stress-relieving support are not sought. Although external stress monitoring oversight and support could be considered infeasible in the current health and social care budget climate, there are examples within the evidence base. For example, Oostra et al [37] describe the co-creation of a digital monitoring tool for the well-being and resilience of family caregivers of people living with dementia with a dashboard monitored by case managers able to provide support.

Participants appreciated stress monitoring techniques that required minimal upkeep and would fit into their daily routines allowing them to focus on their caregiving responsibilities rather than “caring” for the monitoring modality. Minimizing participant burden in any form of digital monitoring and intervention is a priority to retain engagement [38]. However, this is especially imperative in a caregiver population given the need to promote their well-being as an individual within society as well as the possible impact on the caregiving recipient who will also experience negative consequences if their caregiver becomes overwhelmed with stress and develops caregiver strain and burnout [39]. These can include serious consequences such as neglect, mistreatment, and physical and verbal abuse [40,41], which may result in acute hospitalization [42]. Furthermore, the care recipient may need to be transferred to long-term residential care if their family caregiver is no longer able to assume this role [43].

Avoiding inadvertent harm from stress monitoring was vital to our participants again due to the dyadic nature of the caregiving relationship in which inadvertent harm might occur for both the caregiver and the care recipient. A strength of our study is the diversity in our participant sample in terms of age, sex, ethnicity, and relationship to the care recipient. This is particularly important as public and perceived stigma associated with admitting vulnerability and need for support as well as ways of coping with stress are influenced by sex, ethnicity, and relational characteristics [44-46]. Perceived stigma may deter individuals from seeking help and can result in decreased physical and mental health, caregiver burden, lower self-esteem, and disempowerment [47,48]. Care recipient self-perceived burden is common [49]. Given the relational nature of family caregiving that may arise from altruism, reciprocity, a sense of duty, and familial bonds, it makes sense that our participants emphasized that stress monitoring must not increase this self-perceived burden, an aspect that should be prioritized in future studies of interventions designed to address caregiver stress. However, care recipients may consider the need for discretion unimportant and perceive caregiver stress monitoring if accompanied by supportive interventions as beneficial and indeed reduce their sense of self-perceived burden. This is an area requiring further research.

### Recommendations

Our three main findings relating to the measurement of caregiver stress are (1) generating situationally specific data resulting in action to ameliorate stress, (2) not adding to existing caregiver burden, and (3) not inadvertently resulting in harm should be considered by researchers when designing interventions to ameliorate caregiver stress whose effect will be monitored using digital and nontraditional methods. They should also be considered by health care providers, local authorities, and social care providers when considering strategies to understand how best to support family caregiver well-being.

### Limitations

Limitations of our study include a self-selected sample that may have influenced responses. Despite including a demographically diverse sample, the results of this UK study may not be generalizable to other countries, and to non-English speaking cultures. Another limitation is that we did not perform member checking or data triangulation with our study participants to further establish credibility to avoid further time commitment to the study. We did discuss our findings more generally with other former caregivers participating in other areas of our research program.

### Conclusions

In this descriptive qualitative study examining the perspectives of a diverse sample of family caregivers, we found generally favorable impressions of using digital and biosensing technologies to monitor stress. We identified 3 themes that provide useful considerations for the implementation of interventions to ameliorate family caregiver stress of relevance to social care and community teams, researchers, and policy makers. These include stress monitoring that provides meaningful situationally specific data resulting in action, does not contribute to caregiver burden, and does not result in inadvertent harm to either the caregiver or the care recipient.

## Appendix

Multimedia Appendix 1 Consolidated Criteria for Reporting Qualitative (COREQ) checklist.

Multimedia Appendix 2 Interview guide.

Multimedia Appendix 3 Information power.

## Abbreviations

COREQ: Consolidated Criteria for Reporting Qualitative Research
EMA: Ecological momentary assessment
RMT: Remote monitoring technologies

## Data Availability

The datasets generated or analyzed during this study are not publicly available as we do not have permission from the study participants or the research ethics board to make these data publicly available but are available from the corresponding author on reasonable request.
