# Supplementary material for: Family Caregiver Perspectives on Digital Methods to Measure Stress: Qualitative Descriptive Study
Source: J Med Internet Res. 2025 Apr 24;27:e66034. doi: 10.2196/66034 (PMC12062762; doi:10.2196/66034)
Supplement: Multimedia Appendix 2 [file jmir_v27i1e66034_app2.docx]

**Multimedia Appendix #2 – Semi-structured Interview Guide**

1. **What are your thoughts about the importance of measuring caregiver stress?**
2. **Thinking about ways to measure stress of family caregivers, what are your thoughts on how we could do this?**

Probes:

- Thinking about your experiences as a caregiver, what changes do you notice when you are feeling stressed?
- How could these be recorded/monitored?
- Are there specific things (triggers) that might occur, or times when family caregivers are more likely to feel stressed? If yes, can you tell me about those.

1. **Have you ever used technology to support you in your caring role?**

- If yes, what?

1. **What are your thoughts on receiving two or three easy to answer questions about how you are feeling throughout the day on your phone or tablet?**

Probes:

- What questions do you think it would be important to ask to understand how you are coping/how stressed you are feeling?
- When i.e. what time of the day should these questions be asked?
- What would make it easier to answer these questions?
- What would make it more difficult?

1. **What are your thoughts about wearing a device to measure stress such as a fitbit?**

Probes:

- Do you wear one or have you worn one?

*If yes,* can you tell me about why and when you wear one?

*If no,* can you tell me about why you don’t wear one?

- What would encourage you to wear one?

1. **What are your thoughts about wearing another type of sensor to measure stress such as one attached to your skin?**

Probes:

- How do you think it might make you feel to wear a sensor like this?
- What challenges might you expect from wearing a sensor like this?

1. **What concerns would you have about wearing a device to measure stress?**

Probes:

- Costs
- Ethical issues such as equity of access to a device

1. **What are your thoughts on wearing a sensor or answering questionnaires on stress if you are not receiving any help based on the information they provide?**
2. **Is there anything else you would like to share in relation to caregiver stress and how best to measure it?**
