# Supplementary material for: Family Caregiver Perspectives on Digital Methods to Measure Stress: Qualitative Descriptive Study
Source: J Med Internet Res. 2025 Apr 24;27:e66034. doi: 10.2196/66034 (PMC12062762; doi:10.2196/66034)
Supplement: Multimedia Appendix 3 [file jmir_v27i1e66034_app3.docx]

**Multimedia Appendix #3 – Information Power Elements**

| **Study aim** | **Sample specificity** | **Use of established theory** | **Quality of dialogue** | **Analysis strategy** |
| --- | --- | --- | --- | --- |
| Narrow focus on specific strategies (EMA, RMT, fluid sensing) for measuring caregiver stress. Interview questions were specifically directed at these measurement techniques | Highly specific (dense) sample with variability in terms of caregiver age, gender, ethnicity, and relationship to the person being cared | We were aware of established theory in relation to caregiver stress during the design of our study and conduct of our analyses we did not directly apply this theory deductively to our analyses given the narrow focus on use of digital technologies | Strong - Interviews conducted by a researcher with experience and training in qualitative interviews, and with experience working with family caregivers and involvement and interest in digital technologies | Cross-case analysis as we wanted to uncover a range of perspectives in relation to use of technology to measure caregiver stress |
